# A case of marginal zone lymphoma presenting with paraneoplastic vasculitis and pulmonary infiltration

**DOI:** 10.1016/j.rmcr.2025.102362

**Published:** 2025-12-29

**Authors:** Nazli Zeynep Uslu, Umit Yilmaz, Ebru Bilir, Merih Kalamanoglu Balci

**Affiliations:** a Department of Pulmonary Medicine, Bahcesehir University Faculty of Medicine, Istanbul, Turkey; b Department of Family Medicine, Haydarpasa Numune Training and Research Hospital, Istanbul, Turkey

**Keywords:** Marginal zone lymphoma, Paraneoplastic vasculitis, Pulmonary infiltration, Extranodal lymphoma, Case report

## Abstract

**Background:**

Paraneoplastic vasculitis is a rare but important manifestation of underlying malignancies, most commonly hematological, and often presents with atypical features resistant to standard immunosuppression. Marginal zone lymphoma (MZL), a low-grade B-cell lymphoma, may infrequently present with pulmonary involvement and paraneoplastic immune phenomena.

**Case description:**

We report a 73-year-old male with a history of COPD who presented with progressive dyspnoea, weight loss, lower limb purpura, and pancytopenia. Initial suspicion included pneumonia and COPD exacerbation; however, imaging revealed bilateral pulmonary infiltrates and pleural effusion. Laboratory evaluation showed systemic inflammation and worsening cytopenias. Further workup, including PET-CT and biopsies, identified CD20-positive extranodal marginal zone lymphoma with pulmonary infiltration. The patient developed presumed paraneoplastic vasculitis involving the skin and coronary arteries, in the absence of classical autoimmune markers. Rituximab monotherapy was initiated, resulting in both clinical and radiological improvement.

**Conclusions:**

This case highlights the importance of considering underlying malignancy in patients with unexplained systemic inflammation, vasculitic skin lesions, or cytopenias, particularly when autoimmune markers are negative. Prompt histological diagnosis and multidisciplinary collaboration enabled timely immunochemotherapy, resulting in the resolution of both lymphoma and associated paraneoplastic complications.

## 1 Introduction

Systemic vasculitis is most often linked to autoimmune diseases, but in some cases, it can occur as a paraneoplastic response, especially in patients with lymphoid malignancies. Paraneoplastic vasculitis accounts for a small fraction of vasculitic presentations and is most often linked with haematological malignancies, as in non-Hodgkin lymphomas and myelodysplastic syndromes [1]. These vasculitides often present atypically, lack classical autoantibodies, and demonstrate poor response to conventional immunosuppression unless the underlying malignancy is addressed [2].

Marginal zone lymphoma (MZL) is a low-grade B-cell lymphoma that typically arises in mucosa-associated lymphoid tissue but may present in nodal or extranodal forms. Pulmonary involvement is rare, occurring especially in the elderly [3]. It may mimic infection, interstitial lung disease, or even vasculitis radiologically. Moreover, extranodal MZL may give rise to paraneoplastic immune manifestations, such as cytopenia, purpura, and neuropathy.

Cardiac involvement of lymphoma usually presents as coronary vasculitis and is challenging to diagnose. In a previous literature review, the incidence ranges from 8.7 % to 20 % [4]. Inflammatory coronary ectasia without atherosclerosis, as in this case, may be a manifestation of systemic vasculitis or direct paraneoplastic vascular injury.

We report the case of a 73-year-old man with respiratory symptoms initially misattributed to chronic obstructive pulmonary disease (COPD) exacerbation and pneumonia, who was ultimately diagnosed with CD20-positive extranodal MZL a with lung infiltration and probable paraneoplastic vasculitis affecting the skin and coronary vasculature. In older adults with unclear systemic symptoms, keeping a broad differential and involving other teams early can be crucial to reaching the correct diagnosis and starting effective treatment.

## 2 Case presentation

A 73-year-old male with a history of COPD (ex-smoker of 30 packages per year), hypertension, and a prior nephrectomy (due to complicated nephrolithiasis), attended the pulmonary outpatient clinic on December 10, reporting worsening shortness of breath, fatigue, dry mouth, and reduced physical activity (with a prior EMG result of sensorimotor polyneuropathy) over the past month. He had unintentionally lost 7 kg over the last 3 months.

On physical examination, bilateral basal crepitations and wheezing were noted, along with bilateral lower extremity pitting oedema and palpable purpura extending below the knees. Vitals included a blood pressure of 132/80 mmHg, a heart rate of 70 beats per minute, and a respiratory rate of 17 breaths per minute. Oxygen saturation was 96 % as measured by pulse oximetry.

**Fig. 1:**
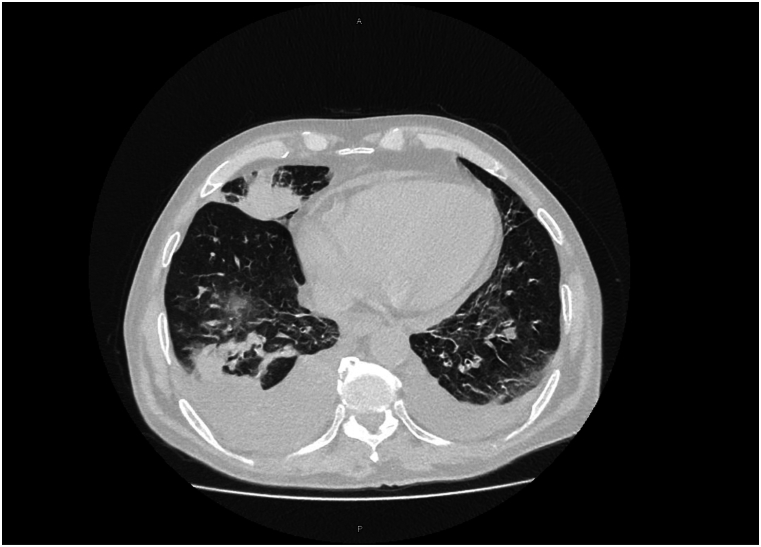
Right-sided middle and lower lobe infiltrates, bilateral lower lobe fibrotic changes, and bilateral pleural effusion.

A thoracic Computed Tomography (CT) scan revealed right-sided middle and lower lobe infiltrates, bilateral lower lobe fibrotic changes, and bilateral pleural effusion (Fig. 1). Laboratory work-up showed elevated CRP and urea, pancytopenia, hypoalbuminemia and worsening renal function (Table 1). The patient was admitted on December 10 with a working diagnosis of pneumonia superimposed on COPD and for further evaluation of possible hematologic and rheumatologic conditions due to systemic symptoms and cutaneous findings.

**Table 1:** Laboratory results on the day of admission.

	Normal Range	Day of Admission
White Blood Cell Count	3.98–10.2 K/uL	3,46 K/uL
Hemoglobin	14.1–18.1 g/dL	8,1 g/dL
Platelet Count	142–424 K/uL	64 K/uL
Neutrophils	1.78–5.38	2,98 K/uL
C-Reactive Protein	0.-8.2 mg/L	112,60 mg/L
Lactate Dehydrogenase	135–225 U/L	133 U/L
Blood Urea Nitrogen (BUN)	18–55 mg/dL	122 mg/dL
Creatinine	0.7–1.3 mg/dL	2,02 mg/dL
Albumin	3.4–4.8 g/dL	2,7 g/dL

On the day of admission, the patient was initiated on supplemental oxygen therapy via nasal cannula at a flow rate of 2 L/min and started on theophylline 200 mg IV once daily, moxifloxacin 400 mg IV once daily, and piperacillin–tazobactam 2.25 g IV three times daily for empirical coverage. Nebulised bronchodilators were organized as ipratropium bromide 0.5 mg four times daily and budesonide 0.25 mg twice daily.

Due to the progression of respiratory distress, including depletion of sO2 and decreased blood pressure, the patient was transferred to the intensive care unit (ICU) from the third to the fifth day of admission for close monitoring and support with non-invasive ventilation. Bilateral pleural drains were also inserted with ultrasound guidance. The pleural fluid appeared transudative in character and was biochemically consistent with a transudate.

Hematologic parameters revealed pancytopenia, prompting haematology consultation (Table 1). The peripheral smear demonstrated anisopoikilocytosis and neutrophilic granulation, without blasts. In the context of systemic inflammation, lymphoproliferative suspicion, and worsening cytopenias, further evaluation, including lymph node biopsy was prioritised. Pancytopenia was considered to reflect either bone marrow involvement or paraneoplastic suppression. Although bone marrow biopsy was indicated to further investigate marrow involvement, the patient declined the procedure after counselling. In the meantime, a PET-CT scan was ordered. It demonstrated hypermetabolic lymphadenopathy in the lungs, mediastinum, abdomen, and cervical regions. Transthoracic lung and submandibular lymph node biopsies were performed. A concern for a possible oesophageal and/or gastric malignancy had led to an upper gastrointestinal endoscopy, but the biopsy results had been negative for neoplasia. Following ICU discharge, erythrocyte suspension transfusion was administered on the sixth day due to persistent anaemia.

Additionally, the presence of purpuric lesions on the legs and pancytopenia prompted evaluation for systemic vasculitis. Autoimmune workup (Table 2) revealed negative ANCA, ENA, antiphospholipid antibodies, and cryoglobulin. ANA was positive with a centromere and cytoplasmic reticular pattern (AC-3 and AC-21), which may occur in non-specific contexts and was not considered diagnostic of a connective tissue disease.

**Table 2:** Serological test results.

	Serological Test Results
Anti-Cardiolipin Antibody IgM	Negative
Anti-Cardiolipin Antibody IgG	Negative
Antinuclear Antibody	Positive 1/320 AC-3 Centromere and AC-21 AMA
Anti-Neutrophil Cytoplasmic Antibodies	Negative
Anti-Cyclic Citrullinated Peptide Antibody	Negative
Anti-Mitochondrial Antibody subtype M2	Positive
Anti-beta-2-Glycoprotein I IgM	Negative
Anti-beta-2-Glycoprotein I IgG	Negative
Extractable Nuclear Antigens	Anti-ENA Sentromer (CENP B) Antibody borderline, others negative
Lupus Anticoagulant	Negative

The finding of isolated anti-AMA-M2 positivity, although not initially prioritised, may indicate a subclinical autoimmune biliary process or overlap syndrome (Table 2). Further hepatological evaluation was planned with gastroenterology in the outpatient clinic.

Submandibular lymph node biopsy demonstrated CD20-positive MZL (extranodal MALT type). Concurrently, transthoracic lung biopsy revealed atypical small B-lymphoid cells consistent with lymphomatous pulmonary infiltration. Given the multisystemic features suggestive of a paraneoplastic vasculitic process, rheumatology and haematology were both involved in the case. Pulse corticosteroid therapy (methylprednisolone IV 500 mg for 3 days) was initiated under respiratory supervision and continued with a gradual taper.

During his hospital stay, inflammatory and biochemical parameters improved steadily. On the eleventh day of admission, CRP had decreased from 288 mg/L to 11.9 mg/L. Serum creatinine fell from 2.02 to 1.11 mg/dL, and albumin rose from 2.2 to 3.1 g/dL (Table 3). These changes reflected a general improvement in his condition. A mild rise in white cell count and ongoing erythrocyte transfusions supported his clinical stability.

**Table 3:** Laboratory results on the day of discharge.

	Normal Range	Day of Discharge
White Blood Cell Count	3.98–10.2 K/uL	6,55 K/uL
Hemoglobin	14.1–18.1 g/dL	8,7 g/dL
Platelet Count	142–424 K/uL	189 K/uL
Neutrophils	1.78–5.38	5,64 K/uL
C-Reactive Protein	0.-8.2 mg/L	11,90 mg/L
Blood Urea Nitrogen (BUN)	18–55 mg/dL	109 mg/dL
Creatinine	0.7–1.3 mg/dL	1,11 mg/dL
Albumin	3.4–4.8 g/dL	3,5 g/dL

On the thirteenth day of admission, the patient was discharged from the respiratory ward with a significantly improved pulmonary status, including the resolution of respiratory distress, a reduced oxygen requirement, and regression of bilateral lower limb purpura.

Given the patient's systemic findings and radiological features, after serological work-up has been done based on a multidisciplinary agreement rituximab monotherapy (375 mg/m^2^ IV) was initiated, He received the first dose on January 6 and a second on January 30. Serial blood tests showed gradual improvement of hematologic parameters, and the patient has remained clinically stable under ongoing outpatient follow-up.

On review dated January 9, the patient reported intermittent chest pain. ECG findings were unremarkable, with stable vital signs. Coronary angiography showed diffuse ectasia involving all three major coronary vessels—RCA, LAD, and circumflex—in the absence of significant atherosclerotic plaque. Given the active systemic disease, a diagnosis of coronary vasculitis was considered. Medical therapy was commenced with metoprolol 25 mg, acetylsalicylic acid 81 mg, and ranolazine 375 mg twice daily.

A follow-up thoracic CT imaging was scheduled to assess post-treatment resolution of parenchymal involvement. Repeat imaging subsequently demonstrated partial regression of pulmonary infiltrates and pleural effusion. The patient's cutaneous vasculitic lesions resolved, and systemic inflammatory markers declined steadily.

On repeat imaging performed ahead of the second rituximab cycle (January 10), the PET-CT revealed encouraging regression: lymphadenopathies in the cervical, mediastinal, and abdominal chains had diminished in both volume and FDG avidity. Pulmonary changes had also partially resolved.

## 3 Discussion

The patient's respiratory symptoms initially resembled a COPD exacerbation due to pneumonia. These findings led to hospital admission and empirical therapy. However, additional systemic features—including peripheral purpura, significant weight loss, and pancytopenia—prompted an expanded diagnostic approach that revealed an underlying haematological malignancy with probable paraneoplastic vasculitis [5].

Histopathology confirmed CD20-positive marginal zone lymphoma (extranodal MALT type) on lymph node biopsy, while lung biopsy also showed lymphomatous infiltration. The presence of systemic inflammatory findings and cutaneous vasculitis, in the absence of primary autoimmune markers supported a diagnosis of lymphoma-associated immune dysregulation rather than primary vasculitis [6,7]. This distinction is essential, as paraneoplastic vasculitis often lacks classical serological markers, such as ANCA or ANA, which was the case in our patient, and typically improves with treatment of the underlying malignancy.

Coronary artery involvement further complicated the clinical picture. Cardiac evaluation revealed diffuse coronary ectasia in all three major vessels, without signs of obstructive atherosclerosis. In the context of elevated troponin and systemic inflammation, coronary vasculitis was suspected. Echocardiography excluded significant ventricular dysfunction. Despite the biochemical evidence of myocardial stress, the patient remained clinically stable and was managed conservatively with beta-blockers, aspirin, and ranolazine.

Although a bone marrow biopsy was not performed, the pancytopenia was considered likely related to either marrow involvement or immune suppression in the context of systemic inflammation. The patient was offered the procedure but chose not to proceed. Rituximab monotherapy was started following discussion among the treating teams [8]. His condition improved with the resolution of respiratory symptoms, skin findings, and a drop in inflammatory markers. Follow-up PET-CT showed both structural and metabolic improvement.

In patients—especially older adults—who present with both respiratory symptoms and systemic findings, it is crucial to consider a broad range of possible diagnoses. In this case, early input from multidisciplinary teams and timely tissue sampling helped identify the cause and direct treatment. The favourable response to rituximab supports the role of targeted immunotherapy in managing not only the malignancy but also its associated paraneoplastic complications.

## 4 Conclusion

Diagnosing paraneoplastic syndromes can be particularly difficult in older patients who present with signs affecting multiple organ systems. In this case, marginal zone lymphoma resembled a common respiratory illness, while also giving rise to immune-related complications such as skin and coronary involvement, despite negative autoimmune tests. The diagnosis was reached through a combination of histology and input from several specialities, which allowed treatment with rituximab to begin promptly. The patient responded well, with improvement in both lymphoma-related symptoms and presumed paraneoplastic effects. In patients with unexplained cytopenias, purpura, or organ dysfunction, especially when typical infections do not fully explain the picture, clinicians should consider an underlying malignancy.

## 5 Patient perspective

The patient was unable/unwilling to provide a perspective for publication.

## CRediT authorship contribution statement

**Nazli Zeynep Uslu:** Conceptualization, Data curation, Formal analysis, Investigation, Methodology, Project administration, Resources, Software, Supervision, Validation, Writing – original draft, Writing – review & editing. **Umit Yilmaz:** Conceptualization, Data curation, Formal analysis, Methodology, Visualization, Writing – original draft, Writing – review & editing. **Ebru Bilir:** Conceptualization, Data curation, Formal analysis, Investigation, Methodology, Project administration, Resources, Validation, Visualization, Writing – original draft, Writing – review & editing. **Merih Kalamanoglu Balci:** Conceptualization, Data curation, Formal analysis, Funding acquisition, Investigation, Methodology, Project administration, Resources, Software, Supervision, Validation, Visualization, Writing – original draft, Writing – review & editing.

## Ethical statement

Written informed consent was obtained from the patient for publication of this case report and any accompanying images.

## Declaration of competing interest

The authors declare that they have no known competing financial interests or personal relationships that could have appeared to influence the work reported in this paper.
